# Bull-bull mounts in Holstein bulls: a survey of its incidence, possible causes, and consequences in Uruguayan bull’ breeders

**DOI:** 10.1590/1984-3143-AR2020-0032

**Published:** 2021-05-28

**Authors:** Stefanie Fajardo, Rodolfo Ungerfeld

**Affiliations:** 1 Departamento de Biociencias Veterinarias, Facultad de Veterinaria, Universidad de la República, Montevideo, Uruguay

**Keywords:** buller syndrome, cattle, dairy cattle, breeding bulls

## Abstract

Bulls frequently display male-male mounts, with consequences in the incidence of injuries, and possibly affecting the reproductive performance of the bulls. This behavior is known as the “buller syndrome” when appears in steers, with several individuals mounting one or a few penmates. The study aimed to collect information on the incidence of bull-bull mounts, the possible associated factors, the productive consequences, and management applied by distributing a survey to Holstein bull breeders in Uruguay. A survey was applied and responded by most Holstein breeders in Uruguay (30/33). Nineteen of the 30 breeders observed mounting behavior among bulls, and 15 of them considered it as a relevant problem. The breeders that observed the behavior had a greater number of bulls than those that did not observe it (P = 0.002). All of them observed that mounts were persistently directed towards the same individual (considering a specific period, while it remained in the group). Of these, 11 (58%) considered that this stopped only when the “buller” bull was removed from the group, mentioning that the behavior was frequently redirected to another individual. The mounts between bulls are a major problem in the breeding of Holstein bulls, with important consequences on weight gain and animal health, reproductive problems such as low libido and seminal quality, and even provoking the death of animals. Although not all breeders reported the existence of the problem, those with the bigger herds did. While some management and/or environmental conditions seem to influence (higher density, regrouping, managements that involve movement of animals, and spring) the incidence of bull-bull mounts, there are no standardized managements to avoid this behavior. Considering that most breeders were interested in including practices to minimize this problem if available, it would be essential to understand better the causes and predisposing factors to decrease its negative impacts.

## Introduction

Males of different mammal species, including most ruminants, frequently display homosexual behaviors, including male-male mounts (see review: Freitas-de-Melo et al., 2014). In this context, bull-bull mounts are frequently observed. However, although it is generally assumed that it has an important impact in breeding and production, direct consequences in the incidence of injuries, and affects the reproductive performance of the bulls, it is striking the scarce knowledge about its causes and consequences. This behavior is also frequent in steers, being known as “buller syndrome” (Voyles et al., 2004). The “buller syndrome” is characterized by persistent and excessive mounts of some individuals (“riders”) towards one or a few penmates (“bullers”) (Clavelle, 2002). In these animals, persistent mounting has negative physical, physiological and psychological effects, in addition to negatively affecting health, productivity (Wettemann and Lehman, 1997), and meat quality (Mohan Raj et al., 1991). Considering the psychological and health consequences, the “buller syndrome” is a main problem in the welfare of confined steers (Tucker et al., 2015). As it cannot be reproduced experimentally under controlled conditions (Schwartzkopf-Genswein et al., 2019), most of the information related to this syndrome has been obtained from animals in feedlots, where its incidence is higher than what can be normally expected (Wettemann and Lehman, 1997). Furthermore, despite being a frequent behavior, there is almost no scientific information available of its occurrence in bulls.

The “buller syndrome” causes significant economic losses in steers, as repeated mounts cause injuries of different types, animal mortality, a decrease of weight gain, and affect the quality of meat of buller individuals (see review: Blackshaw et al., 1997). Displaying this behavior also has energetic costs for animals involved, which also disturbs other individuals in the pen, thus reducing their resting behavior and increasing activity, decreasing the productive performance. Therefore, it is important to determine which factors are related to its incidence. It is known that its frequency is higher in bulls than in steers and that it increases after regrouping animals (Tennessen et al., 1985). It has also been reported that the incidence increases when steers are regrouped in slaughterhouses (Bartoš et al., 1988; Franc et al., 1988). The frequency is greater in large groups of feedlot steers than in small groups and increases when animal density is higher (Acosta et al., 1981). In addition to regrouping, the incidence among steers also increases after modifying management routines (Brower and Kiracofe, 1978), and is higher in summer and/or autumn than in the other seasons (Pierson et al., 1976; Brower and Kiracofe, 1978). Although some authors suggested that it would be linked to hierarchical relationships between individuals (Klemm et al., 1983), this is not demonstrated. Furthermore, in rams (Ungerfeld et al., 2007; Ungerfeld and González-Pensado, 2008) and bison (Vervaecke and Roden, 2006), male-male mounts are not related to social hierarchies when the effects of age are disaggregated.

Overall, the scientific information available about the “buller syndrome” is very scarce, and most has been collected in steers. This strongly limits the possibility of having experimental models, because there is no prior knowledge allowing expecting predictable results. Therefore, the objective of this study was to collect information on the incidence of bull-bull mounts, the possible associated factors, the productive consequences, and the management applied by breeders through a survey to Holstein bull breeders in Uruguay.

## Methods

### Breeders and survey

A survey was applied to Holstein breeders considering those that made bull inscriptions in the period from January 2015 to January 2019 in the genealogical records bases of the Rural Asociación Rural del Uruguay. Overall, these records included 54 producers, with 8,070 bulls. From the original list, only those that registered a minimum of eight bulls during that period were included; however, breeder associations were not considered as the animals are raised in individual farms. Therefore, finally, there were 33 breeders, and 30 of those breeders responded to the survey by phone.

The survey included four blocks of questions, including the characterization of the breeder, the circumstances in which the behavior is observed and the characteristics of the animals involved, the observation of injuries as a consequence, and the managements used to decrease or avoid the incidence of the behavior. The survey was first tested with two Jersey breeders, adjusted, and then applied by phone to the breeders of the Holstein database. It included 25 multiple-choice or open short-answer questions, organized into a first part characterizing the breeder, the herd, and the method of rearing/managing bulls, and a second part related to the identification or not of the “buller syndrome”, regarding the characteristics of the “bullers” and “riders” individuals, the injuries observed and the factors that could influence their incidence.

### Characteristics of the surveyed population

The 30 surveyed breeders were distributed in six Departments (local political regions) located in the south of the country: Canelones (3), Colonia (10), Flores (1), Florida (6), San José (7), and Soriano (3). At the time of the survey, these producers included a total of 265, 514, 90, 174, 155, 110 bulls in each of these Departments, respectively (total = 1308 bulls).

The breeding system varied widely between breeders, with some of them raising based on natural pastures, others based in improved pastures, or paddocks with daily supplementation, with a density of 0.7 to 30 bulls/ha. Besides, in some cases the bulls were bred in the same paddocks with other categories, so the bull density was 0.05/ha.

From the 30 breeders surveyed, 22 bred only Holstein bulls, and the other eight also bred Jersey (5), Angus (1), New Zealand variety (1), and Brown Swiss (1). Overall, 19 did not finish the animals with pen raising, while in the other 11, bulls were raised in pens from birth (3), since they were 4 months old (1), 9 months (1), 15 months (2) or only when the auction is approaching (1). Nine of the 30 breeders surveyed carried out their annual auction.

The frequency of the visits of the breeder or the person in charge of the area where the bulls were varied widely, with two (10.5%) doing two daily visits, 11 (57.9%) one daily visit, 4 (21.1%) three visits/week, and two visits/week (10.5%). Several breeders had bulls in more than one paddock, so there were 8paddocks with less than five bulls, seven with 5-9, five with 10-19, six with 20-29, six with 30-50, and three paddocks with more than 50 bulls. Twenty-six of the 30 breeders (87%) regrouped bulls throughout the breeding period.

### Statistical analysis

Frequencies were compared with the Fishers’ exact probability test and the numerical variables with Anova.

### Ethical approvement

The article does not include the ethical approval for studies with animals because it does not include direct work with animals.

## Results

### Incidence

Nineteen of the 30 breeders (63.3%) observed mounting behavior among bulls, and 15 of them (79.0%) considered it as a relevant problem. The size of the group was greater in those that observed the behavior than in those that did not observe it (P = 0.002), and the number of them that simultaneously had bulls from other breeds also tended to be greater (P = 0.076) (Table 1). There was no difference in the frequency of breeders that bred them in pens or not (P = 0.12) or regroup animals, which was carried out by 73.3% of the total (Table 1).

**Table 1:** Some characteristics of the management of the breeders that reported that they observed or did not observe male-male mounts among Holstein bulls.

**Breeders that reported male-male mounts**	**Size of the group**	**Density (bulls/ha)**	**Bred other bulls from other breed (%)**	**Pen breeding (%)**	**Regroup animals (%)**
Observed	62.7 ± 12.6	7.6 ± 2.5	3/19 (15.8)	10/19 (52.6)	14/19 (73.7)
Did not observe	8.0 ± 1.3	2.8 ± 0.5	5/11 (45.5)	3/11 (27.2)	8/11 (72.7)
P	0.002	0.18	0.076	0.12	0.33

The 19 breeders (100%) who reported the behavior observed that it was persistently directed towards the same individual (considering a specific period, while it remained in the group). Of these, 11 (58%) considered that this stops only when the “buller” bull is removed from the group, mentioning that the behavior is frequently redirected to another individual. The “riders” bulls were “all the rest of the group” according to 13 breeders, those with the highest libido (eight breeders), the largest (seven breeders) or the most dominant (seven breeders), and those with the best body condition (three breeders) or the most aggressive (two breeders) (more than 100% as each breeder could respond more than one answer). Five breeders did not identify any particular characteristics. Twelve of the 19 breeders did not identify any special characteristic of the “buller” bulls. Others responded that it was the smallest of the group (nine responses), a new individual in the group (seven responses), the weakest (six responses), the one with the poorer body condition (two responses), that with bull-bull mounts aspect (two responses), or the least dominant individual (one response), a feminized individual (one response) or one that did not mate (one response).

According to one breeder, the behavior is observed when the group has at least three animals; three breeders mentioned that it is observed when it has at least five animals, another that is when it has from seven to eight animals, 10 reported that it occurs when it has 10 to 15 animals per group, one that it is observed when the group has 15 to 20 bulls, two that the group should have 20 to 25 bulls, and one reported that it is observed in groups of more than 50 animals. Overall, 84% stated that they did not find a relationship between the size of the paddock and the incidence, but three breeders reported a higher incidence when the pasture is lower and the density higher.

### Influencing factors

While 11/19 breeders (58%) reported that this behavior is more frequently observed in spring, the other eight found no link with the season of the year. No breeder reported links between the incidence of this behavior and the type of food. Nine of the 19 (47.4%) breeders reported that the incidence increases after regrouping animals, or moving and locking them up for sanitation or vaccinations, or selecting animals for sale. Only one breeder reported that he observed a greater number of mounts during the afternoon, without other responses related to daily variation.

From the nine breeders who make their auctions, 4 (44.4%) reported that the mounts are more frequent during the handling for it. Two other breeders reported that both, when auctions are carried out and when semen is collected, the animals remain tied during the day, which influences an increase in the incidence of mounts between bulls when they are released at night.

### Lesions and other consequences

All the breeders that observed the behavior in their herds, observed injuries. Five breeders (26.3%) founded dead “buller” individuals. From all, 17 (89.5%) reported that the “buller” bulls had lower weight gains, 16 (84.2%) observed wounds on legs, 15 (80.0%) found the animals separated, in prolonged decubitus, and 14 (73.7%) animals with myiasis. Furthermore, 11 (57.9%) observed that the animals had shaggy hair, 7 (36.8%) abscesses in the rump region, three (15.8%) animals with diarrhea, and three other ischial peelings. Since some collect semen, four reported that sperm quality decreases. Besides, eight other different consequences were mentioned, including anus injuries, locomotor injuries such as hip dislocation, difficulties in moving, claudication, affections of the forelimbs, bruising, spinal cord injuries, general deterioration, and joint pain.

Six breeders mentioned two specific leg injuries. One was described as “affection and removal of the upper phalanges of the hind limbs, and the second as “bladder in front of the hock” corresponding to synovitis/capsulitis of the tarsal joint. In addition, two breeders also mentioned the observation of penile fractures in “riders” bulls.

In addition to these affectations, some breeders also reported on specific behaviors of the “buller” animal, including jumping wires and hiding in the trees, productive alterations such as failures in their pregnancies, low libido (does not mount), or that in many cases it was returned by the buyer.

### Management practices

Eighteen breeders (94.7%) responded that they would be willing to take management measures if it is shown as useful to decrease the problems provoked by bull-bull mounts. The same number of breeders (18) took management measures when identifying the problem. Seventeen of the breeders that reported the occurrence (89.5%) removed the “buller” bull from the paddock, and the same number carried out specific treatments for the lesions (application of antimicrobial drugs, prevention of myiasis, etc.). The only breeders that did not mention that they retire the “buller” bull responded “*it is preferable to maintain the individual that is being harassed in the group under surveillance, withdrawing him only at imminent risk of death, than to removing him.*” Two breeders decided to withdraw or not the “buller” bull depending on the situation, only when the injuries were serious. None of the breeders consulted removed the “rider” bull, explaining that in the number of animals/proportion it is easier to remove the few “bullers”.

Eight breeders (42.1%) select older individuals when they are going to introduce new animals to a stable group, five (26.3%) declared taking other measures, including the confinement of all animals for 24 h before regrouping them (both, new individual to be introduced and pre-existing group) in a pen without food or drink, spraying them with creolin at the end of this period. Other measures mentioned to prevent mounts induced by regrouping were preadapting the animals during 4 days, maintaining them separated by an electric fence allowing their contact but without physical interaction; always raise them in subgroups separated by wire or in nearby pens so that they have already met each other; ensuring that the new ones to be introduced are more or less the same size (homogeneous) as the pre-existing group; maintaining small groups separated by categories (age/weight), and whenever possible, avoid regrouping and/or increasing the vigilance in the period following regrouping. In addition, three breeders (15.8%) mentioned that the size of the group to be introduced has to be between 30 and 50% of the pre-existing group size. Other breeders mentioned that the group to be introduced should have at least two, three, or four bulls (one mention each), at least five bulls, that has at least five bulls (mentioned by four breeders), seven to eight bulls (one mention), and at least 10 to 15 (four mentions). The other four breeders did not consider the size of the group of bulls to be introduced.

## Discussion

To the best of our knowledge, this is the first report on the incidence of bull-bull mates studied, characterizing some factors that influence its incidence, its consequences, and possible strategies to minimize them. Although the behavior was not directly studied, the information was collected from Holstein bull breeders, covering more than 90% of the local breeders and most of the bulls marketed in the country, so it is possible to affirm that the data represents the view of the local Holstein bull breeders. This information is relevant to raise a problem that has not been systematically studied although it affects most bull breeders, but also farmers that raise non-castrated calves of this breed for fattening and slaughter, given that there is sustained information that they grow faster than castrated animals (Steen and Kilpatrick, 1995; Keane, 2003; Kirkland et al., 2006). Groups of non-castrated male bovines in confinement display a greater number of aggressive interactions (Jago et al., 1999), causing social stress, affecting their weight gain (Blumetto et al., 2015). Besides, and as it was noted by most of the breeders, this problem increases the incidence of health problems, even leading to animal deaths. To the aforementioned economic losses, it should be mentioned that all the management, health treatments, human resources, and the need of reserving empty free paddocks to re-allocate the affected individuals during the “recovery” period increases the costs and alters the normal dynamics of the farm.

The information provided by the breeders confirms that there are several predisposing factors. The number of bulls in the group favors the display of the mounts between bulls, similarly as it was reported in steers (Blackshaw et al., 1997). Although the density did not significantly influence the incidence, it is important not to rule out its possible effect since the number of producers was limited and the density had a variable with high dispersion. Three breeders stated that the higher density of bulls favors the manifestation of mating between bulls of the herds, which is consistent with the reports of the OIE (2009) in which it is stated that a high density modifies social behaviors affecting bovine well-being. The breeders did not report a relationship between diet and the manifestation of behavior, but it cannot be ruled out as also their mother's diet during pregnancy could influence, as Phillips (2002) hypothesized.

One factor that was consistently mentioned as triggering this behavior is the regrouping of bulls. Grouping unknown animals produce an increase in aggressiveness with negative effects on their health and well-being (Patt et al., 2012). The grouping destabilizes the social hierarchy (Andersen et al., 2008), which generates social stress, and in bulls, the increase in aggressiveness is accompanied by an increase in bull-bull mounts (Mohan Raj et al., 1991). Consistently with this, the European Consulate (European Union, 1988) recommends not introducing bulls to previously established groups, since the mixture of animals leads to an increase in aggressiveness during the process in which a new hierarchy is established (Bouissou et al., 2001). In the same sense, in goats, it has been shown that mixing bucks provokes a marked increase in the number of aggressive (Sánchez-Dávila et al., 2018) and sexual interactions (Ungerfeld et al., 2013) in the group. This is also accompanied by increases in cortisol concentration (Giriboni et al., 2015; Sánchez-Dávila et al., 2018), and negatively affects testosterone production, testicular size, and seminal quality for at least 20 days (Giriboni et al., 2015). Although in this survey the seminal quality was not evaluated, some breeders mentioned the negative effect of mounting between bulls on it, as well as on libido, affecting directly even in marketing (return of bulls). Though several breeders mentioned managements that diminish this problem (considering the age of the bulls or that they know each other before), in coincidence with Schwartzkopf-Genswein et al. (2019), no breeder mentioned that these practices eliminate the problem. Furthermore, although some institutions recommend that only animals of similar size and age should be regrouped (EFSA, 2013), to the best of our knowledge no comparative data is available.

Although no breeder proposed it as management to reduce the incidence of the mounts between bulls, the number of breeders who reported its incidence was lower when they also bred together bulls from other breeds. Although according to our knowledge there is no specific information, there are differences in the incidence of behavior between bulls of different breeds, and some breeds may result more frequently in “bullers” roles (Jezierski et al., 1989). In consistence, in general, bulls would be more aggressive towards other individuals of their breed (Jezierski et al., 1989), decreasing then the intensity of aggressiveness in mixed herds.

The higher incidence in spring coincides with what was previously reported in steers (Bozkurt et al., 2006). However, it is not possible to know if this is associated with endocrine changes such as estradiol concentrations (Jezierski et al., 1989; Meyer, 2001), and therefore the aggressive (Jago et al., 1999) and sexual behavior of bulls, or if it is related with changes in feeding, in both quantity and composition. Indeed, Bozkurt et al. (2006) reported that this behavior was more frequent in steers that accessed higher nutritional levels, although in the survey the breeders did not associate the behavior with the type of food.

Although Phillips (2002) stated that at least in the mount between steers there is no penile intrusion, the report by at least two producers of penile fractures indicates that when the mount is between whole bulls the intrusion can occur and even injure the penis. Although this is frequently observed in the breeding of bulls and is reported as a pathway for dissemination of venereal diseases, it was striking that it was not possible to find literature that studies this aspect that has important consequences on the health and well-being of bulls (Phillips, 2002).

## Conclusions

In summary, these data show that the mounts between bulls are a major problem in the breeding of Holstein bulls, with important consequences on weight gain and animal health, reproductive problems such as low libido and seminal quality, and even provoking the death of animals. Although not all breeders reported the existence of the problem, those with the bigger herds did it. While some management and/or environmental conditions seem to influence (higher density, regrouping, and management that involves movement of animals, spring), there are no standardized managements to avoid this behavior, so it is essential to understand better the causes and predisposing factors to decrease its negative impacts. Moreover, most breeders were interested in including practices to minimize this problem if better tools are available. Most reported practices to reduce the incidence of this behavior are related to pregrouping practices, so it should be important to consider the experience of the breeders that could reduce this problem before regrouping bulls.
